# Long non-coding RNA (lncRNA) HOXD-AS2 promotes glioblastoma cell proliferation, migration and invasion by regulating the miR-3681-5p/MALT1 signaling pathway

**DOI:** 10.1080/21655979.2021.1977104

**Published:** 2021-11-21

**Authors:** Xingming Zhong, Yong Cai

**Affiliations:** Department of Neurosurgery, The First People’s Hospital of Huzhou, the First Affiliated Hospital of Huzhou University, Huzhou, China

**Keywords:** Glioblastoma, LncRNA HOXD‐AS2, miR-3681-5p, MALT1

## Abstract

Glioblastoma (GBM) is the most lethal type of brain cancer. An increasing number of studies suggest that long non-coding RNAs (lncRNAs) are implicated in tumor progression. LncRNA HOXD‐AS2 was reported to be highly expressed in glioma and associated with glioma grade and poor prognosis. However, the molecular mechanism remains to be elucidated. In this study, we first analyzed differentially expressed lncRNAs in glioblastoma using RNA-seq dataset (156 GBM samples and 5 adjacent normal samples in TCGA (Cancer Genome Atlas) and GTEx (Genotype-Tissue Expression) database). HOXD-AS2 was found to be significantly up-regulated in GBM tissues, which was further confirmed in GBM patient tumor samples and GBM cell lines. Silencing HOXD-AS2 inhibited cell proliferation, migration and invasion, and promoted cell apoptosis. We further identified and validated miR-3681-5p as a target of HOXD-AS2, and miR-3681-5p was negatively regulated by HOXD-AS2. By negatively affecting miR-3681-5p, HOXD-AS2 could promote the expression of MALT1 to augment the aggressiveness of GBM cells. miR-3681-5p overexpression or MALT1 knockdown attenuated aggressiveness of GBM cells. Importantly, silencing HOXD-AS2 suppressed tumorigenesis of GBM cells in the xenograft mouse model. Collectively, our study clarified the role of miR-3681-5p/MALT1 axis underlying the oncogenic function of lncRNA HOXD-AS2 in GBM. Future work is required to study the mechanism by which HOXD-AS2 is upregulated in GBM cells, which can provide novel insights into therapeutic intervention for GBM treatment.

## 1. Introduction

Glioblastoma (GBM) is the most lethal type of brain cancer. After diagnosis, optimal surgery plus chemoradiotherapy serves as the standard treatment for GBM [1]. However, the average survival time of GBM patients is 12–18 months and only 25% of patients survive more than one year [1]. Identifying novel therapeutic targets in the development, invasion and migration in the progression of GBM is of great importance in improving treatment outcome and the overall survival of GBM patients [2]. Gliomagenesis is frequently accompanied by genomic and transcriptional aberrations, and its molecular pathogenesis remains to be fully elucidated [1,2].

Long non‐coding RNAs (lncRNAs) are a class of endogenous non-coding RNAs with more than 200 nucleotides. Although lncRNAs do not encode functional proteins, they are widely implicated in transcriptional and posttranscriptional regulation [3]. A number of lncRNAs have been characterized as tumor suppressors [4–6]. In addition, accumulating evidence have shown that the dysregulation of lncRNAs has important implications in tumor initiation, invasion and metastasis [7,8], which is associated with the prognosis in cancer patients. LncRNA HOXD‐AS2 was previously reported to be highly expressed in glioma as compared to normal astrocyte cell lines or tissues [9]. Silencing lncRNA HOXD‐AS2 can inhibit the proliferation of glioma cells as well as the tumorigenesis *in vivo*, and its expression level is correlated with the tumor grade and prognosis in GBM [9]. Although a few studies demonstrated the role of HOXD-AS2 in cell cycle regulation and glioma progression [9,10], the molecular mechanism by which HOXD-AS2 modulates the aggressiveness of GBM remain to be fully investigated.

The mucosa-associated lymphoid tissue protein 1 (MALT1) is an intracellular protein responsible for assembling a signaling complex in Nuclear factor kappa B (NF-κB) activation [11], thereby regulating the activity of NF-κB signaling cascade [12,13]. As a paracaspase protease, MALT1 functions to cleave a number of substrates such as A20, RelB, and NF-κB-inducing kinase (NIK), which leads to the activation of NF-κB [12,14]. MALT1 is therefore recognized as an oncogene and a promising therapeutic target for cancers highly dependent on NF-κB activaton [13,14]. Interestingly, a previous study indicates that miR-181d overexpression can sensitize GBM cells to chemotherapeutics by targeting MALT1 and NF-κB signaling pathway [15]. In contrast, miR-3681 was proposed as a negative regulator in the proliferation and migration of cervical cancer cells [16]. Since GBM is a type of cancers associated with aberrant activation of NF-κB pathway [1,15], investigating the micoRNA targets of HOXD-AS2 and its potential role in pathway could provide novel insights for therapeutic intervention.

In this study, we aim to study the potential role of lncRNA HOXD-AS2 in GBM and the underlying molecular mechanism. We first analyzed differentially expressed lncRNAs in glioblastoma using RNA-seq dataset (156 GBM samples and 5 adjacent normal samples in TCGA (Cancer Genome Atlas) and GTEx (Genotype-Tissue Expression) database). HOXD-AS2was found to be significantly up-regulated in GBM tissues, which is consistent with a previous report [9]. The upregulation of HOXD-AS2 was validated in GBM cell lines, and silencing HOXD-AS2 inhibited cell proliferation, migration and invasion, and promoted cell apoptosis. We further identified and validated miR-3681-5p as a target of HOXD-AS2, and miR-3681-5p was negatively regulated by HOXD-AS2. By negatively affecting miR-3681-5p, HOXD-AS2 could promote the expression of MALT1 to augment the aggressiveness of GBM cells. miR-3681-5p overexpression or MALT1 knockdown attenuated aggressiveness of GBM cells. Collectively, our study clarified the role of miR-3681-5p/MALT1 axis underlying the oncogenic function of lncRNA HOXD-AS2 in GBM.

## 2. Materials and methods

### 2.1 GBM expression dataset and patients sample collection

Data from a total number of 156 GBM tumor samples and 5 adjacent normal samples were retrieved from TCGA (Cancer Genome Atlas, http://cancergenome.nih.gov) and GTEx (Genotype-Tissue Expression, https://gtexportal.org/home/) database for lncRNA expression analysis. In addition, analysis of HOXD-AS2 expression was further confirmed from the GBM sample (n = 163) and normal tissue (n = 207) in GEPIA database (http://gepia.cancer-pku.cn/).

This study involving GBM patient sample collection was approved by the Ethics Committee of The First People’s Hospital of Huzhou, Zhejiang, China (HZU-20112-01). All patients gave their written informed consents, and sample collection and sample processing were conducted according to the Declaration of Helsinki principles. Tumor tissues and para-cancerous normal tissues were collected from 92 GBM patient by surgery, which were frozen immediately in liquid nitrogen until further analysis. The clinical pathological parameters of GBM patients enrolled in the study is summarized in Table 1.

**Table 1.** Correlation analysis between HOXD-AS2 expression and the clinical pathological parameters of GBM

Factor		lncRNA HOXD-AS2		P value
		Low (n = 46)	High (n = 46)	
Gender				0.531
	Male	24	20	
	Female	22	26	
Age				0.398
	≤50	19	22	
	>50	27	24	
Lymph nodes metastasis				0.03
	Negative	42	29	
	Positive	4	17	
TNM				0.017
	Stage I	21	9	
	Stage II	13	11	
	Stage III	7	14	
	Stage IV	5	12	
Vascular invasion				0.023
	Negative	41	31	
	Positive	5	15	

The expression of HOXD-AS2 was strongly linked with TNM staging of the tumor, lymphatic metastasis, and vascular invasion (p < 0.05), but not with age, sex, and race.

### 2.2 Cell culture and transfection

U87 MG, A172, LN229 glioblastoma cell line and normal human astrocytes (NHAs) were purchased from American Type Culture Collection (ATCC, Manassas, VA, USA). The cells were cultured in Dulbecco’s Modified Eagle’s Medium (Thermo Fisher Scientific, 21,875,034) containing 10% FBS (Thermo Fisher Scientific, #26,140,087) and 1% penicillin/streptomycin (Hyclone, #SV30010) in a humidified incubator at 37°C with 5% CO2. Cells were harvested for experiments in exponential growth state.

For cell transfection experiment, siRNA for lncRNA HOXD-AS2, lncRNA HOXD-AS2 expression vector, siRNA for MALT1, control siRNA (NC), miR-3681-5P mimic, miR-3681-5P inhibitor and miR-NC were synthesized by GenePharma (Suzhou, China). Cells were seeded in 6-well plates at a density of 5x10^5 cells/well. 24 h later, 200 nM of each molecule or 6 µ expression plasmid was added into 100 µl Opti-MEM® I Reduced-Serum Medium (Invitrogen, Carlsbad, CA, USA, #31,985,062), and then 6 µL Lipofectamine 3000 reagent (Thermo Fisher Scientific, #L3000001) was added for 10 min incubation at room temperature. The mixtures were added to cell culture dropwise. 48 h after transfection, cells were harvested for further experiments.

### 2.3 Quantitative real-time polymerase chain reaction (qRT-PCR)

TRIzol one-step RNA isolation kit (TaKaRa Bio, Dalian, China, Cat. #9108) was used to extract total RNA from tissues and cells according to the instructions. The extracted total RNA was dissolved in DEPC water and its concentration was measured with NanoDorp. 5 μg of total RNA was used for reverse-transcription into cDNA by PrimeScript RT Reagent Kit (TaKaRa Bio, Dalian, China, # RR037B). cDNA synthesis was performed at 37°C for 15 min and the reaction was inactivated at 85°C for 5 sec. The resulted cDNA was diluted and analyzed in a 7500 Real Time PCR System (Applied Biosystems/Life Technologies, Carlsbad, CA, USA) using SYBR premix EX TAQ II kit (TaKaRa, Dalian, China, #RR820A). The PCR cycling condition used: 95°C 2 min, 40 cycles of 95°C 30 sec, 60°C 30 sec and 72°C 60 sec, with signal detection at the end of each cycle. PCR was performed in 20 µL reaction. Finally, the 2^–∆∆Ct^ method was used to analyze the relative gene expression level by normalizing to GAPDH internal reference gene.

All primer sequences were synthesized and purchased from Shanghai Sangon Biotechnology Co., Ltd. (Shanghai, China):

lncRNA HOXD-AS2: F 5ʹ-AGGAACTGCTCTGGTGAACTCC-3ʹ, R 5ʹ- TGGGCATCTCTTTCAGGAAGGT-3ʹ;

miR-3681-5P, F 5ʹ- TAGTGGATGATGCACTCTGT −3ʹ; R 5ʹ- GAACATGTCTGCGTATCTC −3ʹ;

MALT1, F 5ʹ-AGAGAAGTGTTGATGGCGTCT-3ʹ, R 5ʹ- GTAGTGAGGAATAGGGCTTCCA −3ʹ.

GAPDH: F 5ʹ-GGAGCGAGATCCCTCCAAAAT-3ʹ, R 5ʹ- GGCTGTTGTCATACTTCTCATGG-3ʹ.

### 2.4 Kaplan-meier plotter analysis

The GBM patients were divided into lncRNA HOXD-AS2 low expression and high expression group based on the median expression level of HOXD-AS2 detected by qRT-PCR. Kaplan-Meier plotter (KM-Plotter) (http://kmplot.com/analysis/) is used to analyze the overall survival curve of GBM patients between low and high expression groups.

### 2.5 Cell proliferation assay

Cell proliferation was examined by the Cell-Counting Kit 8 (Engreen Biosystem, Beijing, China, #CA1210). Briefly, cells were seeded in to a 96 -well plate at a density of 1000 cell/well and cultured in a humidified cell culture incubator for 0, 24, 48, 72 and 96 h, respectively. Subsequently, 10 µL CCK-8 reagent was added to the cell culture at indicated time point and incubated for 1 h in a humidified cell culture incubator. The light absorption value (OD value) in each condition was captured at 450 nm wavelength on a Synergy H1 microplate reader (Winooski, Vermont, USA).

### 2.6 Wound-healing assay

Cells were seeded in 6 well plates for about 80% confluence. A scratch wound was created using a sterile 200 μL pipette tip in the central region of each well. The cells were incubated at 37°C for 24 h. Cell images were captured using an inverted light microscope (Olympus, Japan). The migration distance was analyzed is using ImageJ software. The migration rate is calculated as ratio of (would distance at 0 h – would distance at 24 h)/ would distance at 0 h.

### 2.7 Cell invasion assay

A Transwell® system (Corning, NY, USA, #3401) coated with Matrigel (BD Biosciences, Bedford, MA# 356,234) was used for invasion assay. 12-μm pore polycarbonate filters of the upper chamber were pre-coated with Matrigel and 5 × 105 cell were inoculated into the transwell upper chamber in serum-free medium. The upper chamber was loaded into the culture well containing 10% serum-containing medium. After 18 h, culture medium was discarded and the cells were fixed with 4% paraformaldehyde at room temperature for 10 min and stained with 0.5% crystal violet (Sigma, Germany, #109,218) for 20 min. Invading cells from five randomly selected fields of view under each condition were imaged and counted under an inverted microscope (Olympus, Japan) [17,18].

### 2.8 Flow cytometry analysis of apoptosis

The analysis of apoptosis was performed using the FITC Annexin V Apoptosis Detection Kit (BD Biosciences, San Jose, CA, USA, #556,547) according to the manufacturer’s instructions. In brief, 5 μL Annexin V-FITC and 5 μL PI were added to the 1000 μL cell resuspension with 1 million cells and 30 min incubation in the dark. Stained cells were centrifuged and washed twice with 1xPBS and resuspended in 400 μL PBS. Flow cytometry analyses were performed on FACSCalibur (BD Biosciences, Cytocell, Nantes, France) and processed using FlowJo software.

### 2.9 Dual luciferase reporter assay

Luciferase reporter assays were performed to determine whether miR-3681-5p functionally interact with lncRNA HOXD-AS2 or MALT1 30-UTR. A wild-type and mutated HOXD-AS2 or MALT1 (MALT1 3ʹ-UTR) sequencing miR-3681-5p binding site was cloned into the PmirGLO vector expressing firefly luciferase respectively (Promega, E1330). The reporter plasmid and Renilla luciferase (hRlucneo) plasmid (control for transfection) were co-transfected with microRNA mimic or negative control into U87 cells at 70% confluence in a 24-well plates. 48 h post transfection, the relative luciferase activities were measured using Dual-Luciferase Reporter Assay Kit (Promega, E1910) on a luminescence microplate reader (Infinite 200 PRO; Tecan). The relative firefly luciferase activity in the reporter plasmid was normalized to that of Renilla luciferase (hRlucneo) control plasmid.

### 2.10 Protein extraction and western blot

Total protein was extracted from cells using RIPA lysis buffer containing protease inhibitor cocktail (Thermo Fisher Scientific, 78,429). Protein concertation was quantified using a BCA Protein assay kit (Beyotime Biotechnology, Shanghai, China, #P0009). A total amount of 20 ug protein was used for SDS-PAGE electrophoresis and was then transferred onto the PVDF membrane (BioRad, Irvine, CA, USA, 1,620,177). After blocking with 5% skimmed milk for 1 h, the membrane was incubated with primary antibodies: MALT1 (1:1000 dilution; Cell Signaling Technologies, Danvers, MA, USA, #2494S), GAPDH (1:2000 dilution; Cell Signaling Technologies, #2118) overnight at 4°C. After washing with TBST buffer, the membrane was further incubated with HRP-linked secondary antibody (1:3000 dilution; Cell Signaling Technologies, #7074) at room temperature for 1 h. After 4 washes with TBST buffer, and the protein bands were developed using an enhanced chemiluminescence kit (Santa Cruz, TX, USA, sc-2048) and photographed on a gel imager system (Bio-Rad, Hercules, CA, United States). The densitometry analysis was performed with Image J software [19].

### 2.11 Xenograft tumorigenesis in nude mice

All animal procedures were approved by the animal care and use ethical committee of the First People’s Hospital of Huzhou, Zhejiang, China (HZU-20112-02). Twelve immunodeficient nude mice (6 weeks old) were randomly divided into two groups (6 mice in each group): (1) NC group (injected with U87 cells infected with siRNA control NC), (2) si-HOXD-AS2 group (injected with U87 cells infected with siRNA for HOXD-AS2). All mice were housed in specific pathogen-free (SPF) conditions at 24°C with a 12-h day-night cycle. 0.2 mL of cell suspension containing 5 × 105 cells was injected into the flank of each mice as described [20]. Tumor volume were monitored every three days for 4 weeks. 4 weeks after tumor cell inoculation, all the mice were euthanized by CO2 asphyxiation. Briefly, a euthanizing chamber was connected to a carbon dioxide cylinder and the flow rate was adjusted to displace 20% of the cage volume per minute. Mice were placed into the euthanizing chamber for 10 min until no movement was observed. Tumor sample were dissected and fixed in formalin until further experiment.

### 2.12 Immunohistochemistry

Immunohistostaining of Ki67 was performed on 4-mm sections of formalin-fixed paraffin-embedded (FFPE) tumor tissue using VENTANA BenchMark Special Stain platform (Roche, Indianapolis, IN, USA). Briefly, the section was dehydrated and antigen unmasking was performed by heating the section submersed in 1X citrate unmasking solution (SignalStain® Citrate Unmasking Solution, Cell Signaling Technologies, #14,746) for 10 min at a sub-boiling temperature (95°-98°C). After cooling, sections were washed in dH_2_O three times and then incubated in 3% hydrogen peroxide for 10 min. The section was blocked 1 h at room temperature in TBST buffer with 5% Normal Goat Serum, and then incubated with primary antibody anti-Ki-67 (D3B5) (Cell Signaling Technologies, #9129) diluted 1:500 in SignalStain® Antibody Diluent (#8112) overnight at 4°C. After 3 washes with TBST buffer, the section was incubated with HRP-conjugated secondary antibody (1:3000 dilution; Cell Signaling Technologies, #7074) for 60 min at room temperature. After wash, the section was stained by a diaminobenzidine staining kit (ZSGB-BIO, Beijing, China) for 30 min at room temperature. The section was washed in dH_2_O two times and then dehydrated. Section was mounted with coverslips using the mounting medium (Cell Signaling Technologies, #14,177) before imaging.

### 2.13 Statistical analysis

Data are presented as ± standard deviation (SD) from at least three independent experiments. Statistical analyses were performed with SPSS 20.0 software (IBM SPSS, Armonk, NY, USA). Anderson–Darling test was used to test the data normality distribution. The statistical difference between two groups was compared using unpaired student’s t tests. Comparisons among multiple groups were analyzed using one-way analysis of variance (ANOVA) with Tukey’s post hoc test for pairwise comparison. Comparisons of data at multiple time points were examined using two-way ANOVA. P < 0.05 was considered to be statistically different. For survival analysis, the median of HOXD-AS2 expression of the GBM tumor samples was used as cutoff to divide 92 patients into high expression (n = 46) and low expression (n = 46) groups. Kaplan Meier Curve and log-rank test were used to compare the cumulative survival rates. Chi-square statistics was calculated to analyze the correlations between HOXD-AS2 level and the clinical pathological parameters of GBM patients. Spearman correlation was used to analyze the correlation between MALT1, HOXD-AS2 and miR-3681-5p expression in GBM tumor samples.

## 3. Results

In this study, we aim to study the potential role of lncRNA HOXD-AS2 in GBM and the underlying molecular mechanism. We found that HOXD-AS2 was significantly upregulated in GBM tissues, which was validated in GBM patient tumor samples and cell lines. Silencing HOXD-AS2 inhibited cell proliferation, migration and invasion, and promoted cell apoptosis. We further identified miR-3681-5p as a target of HOXD-AS2, and miR-3681-5p was negatively regulated by HOXD-AS2. By negatively affecting miR-3681-5p, HOXD-AS2 could promote the expression of MALT1 to augment the aggressiveness of GBM cells. Collectively, our study clarified the role of miR-3681-5p/MALT1 axis underlying the oncogenic function of lncRNA HOXD-AS2 in GBM.

### 3.1 LncRNA HOXD-AS2 expression is significantly up-regulated in GBM tissues and cell lines

**Figure 1.**
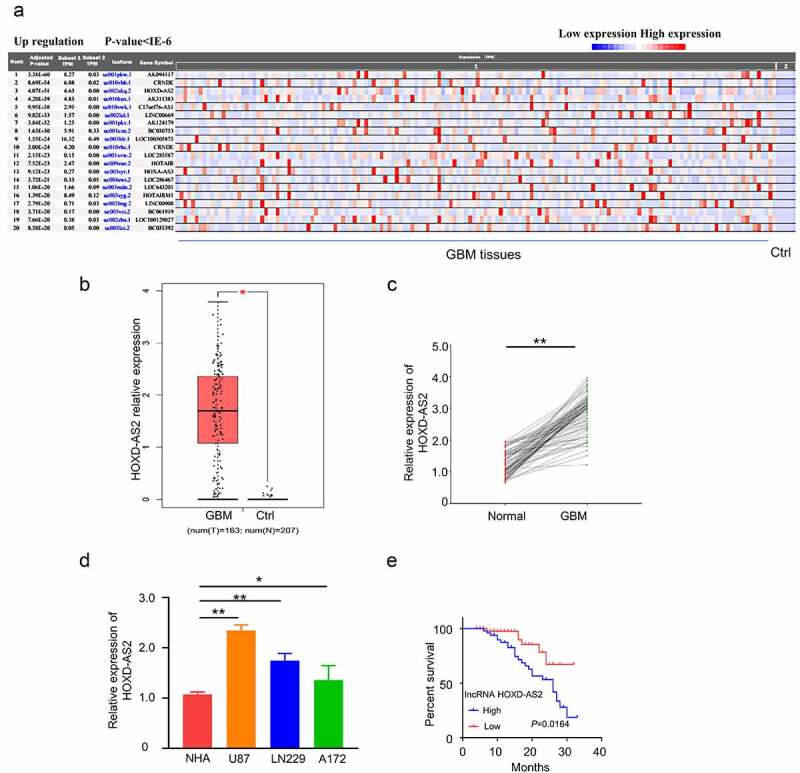
LncRNA HOXD-AS2 expression is significantly up-regulated in GBM tissues and cell lines

We first analyzed the differentially expressed lncRNAs in glioblastoma of the publicly available RNA-seq dataset (156 tumor samples and 5 adjacent normal samples) in the TCGA and GTEx database. Consistent with a previous report [9], HOXD-AS2 was remarkably upregulated in tumor tissues (Figure 1a, *p* = 4.07E-51). In addition, analysis of HOXD-AS2 expression in the GBM sample (n = 163) and normal tissue (n = 207) in GEPIA database also revealed the significant upregulation of HOXD-AS2 in tumor tissues (Figure 1b). We further compared HOXD-AS2 expression in 92 pairs of GBM patient GBM patient sample and the adjacent normal tissues by qRT-PCR, which validated the upregulation of HOXD-AS2 in GBM tumor tissues (Figure 1c). Besides, the expression level of HOXD-AS2 in U87, A172, and LN229 cell line of GBM was significantly higher than in normal human astrocytes (NHAs) (Figure 1d). We further compared the overall survival between HOXD-AS2 high and low expression patients by Kaplan‐Meier (KM) plotter analysis. Patients with high expression of HOXD-AS2 was associated with a poorer prognosis (Figure 1e). Chi-square test demonstrated that a high expression level of HOXD-AS2 was also significantly associated with TNM staging of GBM tumor, lymphatic metastasis and vascular invasion, but not with gender or age (Table 1). Collectively, the above data suggest that the upregulation of HOXD-AS2 may contribute to the progression of GBM.

(A) According to the TCGA (Cancer Genome Atlas) and GTEx (Genotype-Tissue Expression) database, a total number of 156 tumor samples and 5 adjacent normal samples were included for lncRNAs expression analysis. HOXD-AS2 was remarkably upregulated in tumor tissues. (B) Through GEPIA database (GBM sample (n = 163) and normal tissue (n = 207)), HOXD-AS2 was also significantly increased in tumor tissues than normal tissues. (C) qRT-PCR showed that the expression of HOXD-AS2 in GBM tumor tissues was significantly upregulated than in adjacent normal tissue. (D) The expression of HOXD-AS2 in U87, A172, and LN229 cell lines of GBM was significantly higher than in normal human astrocytes (NHAs). (E) Kaplan‐Meier (KM) plotter analysis showed that patients with high expression of HOXD-AS2 were associated with poorer prognosis. The above data in C-D are the summary of the measurements of 3 independent experiments (mean ± standard deviation). *, P < 0.05, and **, P < 0.01.

### 3.2. Silencing HOXD-AS2 inhibits GBM cell proliferation, migration and invasion, and promotes cell apoptosis

**Figure 2.**
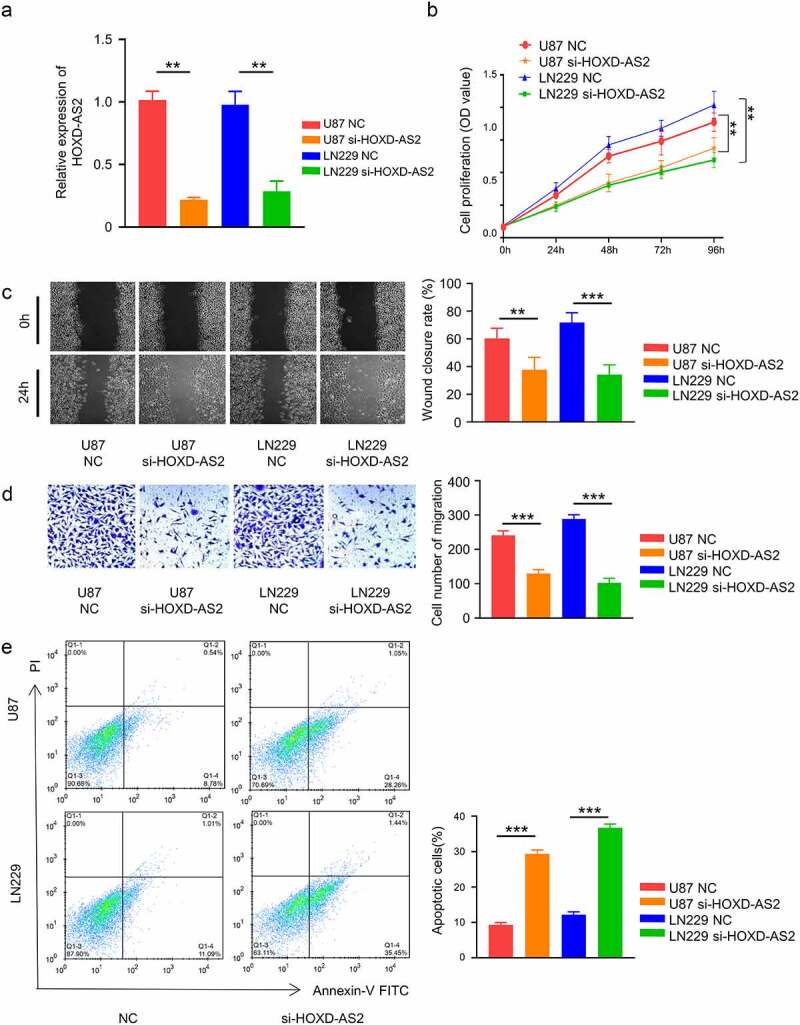
Knockdown of HOXD-AS2 inhibits GBM cell proliferation, migration and invasion, and promotes cell apoptosis

To investigate the functional implication of HOXD-AS2 in GBM cells, we applied siRNA targeting HOXD-AS2 in 2 GBM cell lines with high expression of HOXD-AS2 (U87 and LN299). Transfection of HOXD-AS2 siRNA significantly reduced HOXD-AS2 expression level in both cell lines (Figure 2a). CCK-8 cell proliferation assay demonstrated that silencing HOXD-AS2 significantly suppressed the proliferation of both U87 and LN299 cell lines (Figure 2b). Besides, in both wound-healing assay and transwell invasion assay, HOXD-AS2 knockdown significantly inhibited cell migration and invasion ability the migration ability (Figure 2c and 2d). We further performed apoptosis assay using Annexin V and PI staining. Our results showed that HOXD-AS2 knockdown the increased the percentage of apoptotic cells (Figure 2e). Therefore, these results indicate that HOXD-AS2 is indispensable for the aggressive phenotype of GBM cells.

(A) U87 and LN229 cells with high expression of HOXD-AS2 were used to perform si-HOXD-AS2 knockdown experiment. The qRT-PCR result demonstrated that si-HOXD-AS2 significantly reduced HOXD-AS2 expression. (B) CCK-8 cell proliferation assay indicated that HOXD-AS2 silencing significantly decreased cell proliferation. (C) Wound-healing assay showed that HOXD-AS2 silencing significantly decreased the migration ability. (D) Transwell invasion assay showed that the HOXD-AS2 silencing impaired the invasion ability. (E) FACS analysis of Annexin V and PI staining showed that HOXD-AS2 silencing promotes apoptosis. The above data are the summary of the measurements of 3 independent experiments (mean ± standard deviation). **, P < 0.01, and ***, P < 0.001.

### 3.3. HOXD-AS2 negatively interacts with miR-3681-5p

**Figure 3.**
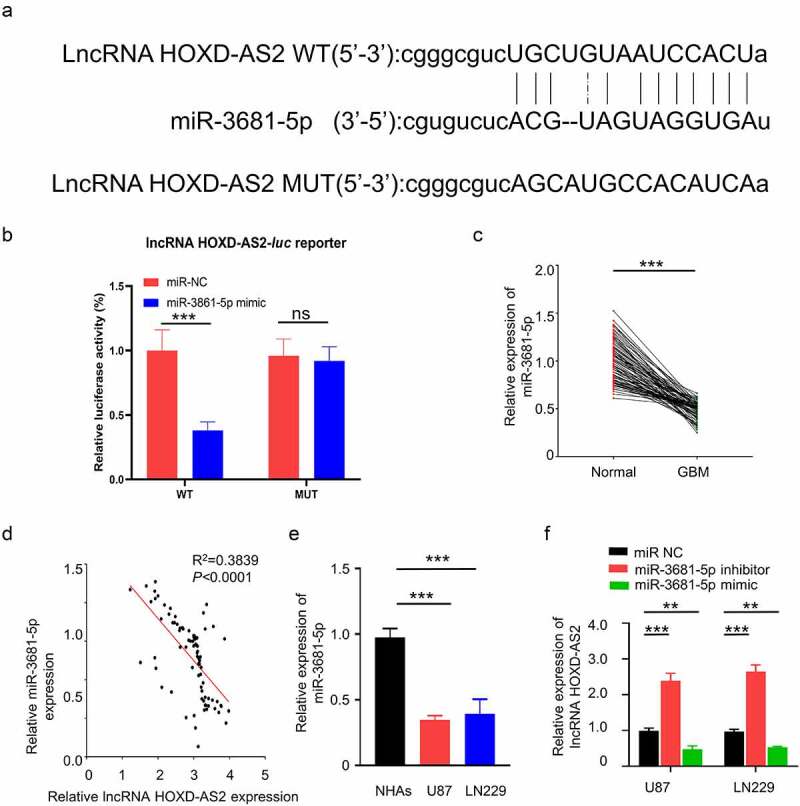
HOXD-AS2 acts as a sponge for miR-3681-5p

To search for the downstream target of HOXD-AS2, we used Starbase 2.0 database (http://starbase.sysu.edu.cn/) to predict the miRNAs containing potential binding site to HOXD-AS2. We found that miR-3681-5p shared a complementary sequence with HOXD-AS2 3ʹ UTR (Untranslated Region) region (Figure 3a). To validate the functional interaction between HOXD-AS2 and miR-3681-5p, we performed dual luciferase reporter assay using reporters containing HOXD-AS2 wild type 3ʹ UTR (WT) or mutated 3ʹ UTR (MUT) in U87 cells. As compared with miR-NC, the transfection of miR-3681-5p mimic inhibited luciferase activity of the WT reporter, which was abrogated in the MUT reporter (Figure 3b). Additionally, qRT-PCR analysis showed that miR-3681-5p was significantly downregulated in GBM patient tumor samples, and its expression was negatively correlated with HOXD-AS2 expression level in GBM tumor samples (Figure 3c and 3d). In comparison to NHA cells, the expression level of miR-3681-5p in GBM cell lines of U87 and LN229 was significantly lower (Figure 3e). Additionally, transfection of miR-3681-5p inhibitor caused significant upregulation of HOXD-AS2 expression while miR-3681-5p mimic decreased HOXD-AS2 expression (figure 3f). The above results suggest that HOXD-AS2 negatively interacts with miR-3681-5p.

(A) By the analysis in Starbase 2.0 database (http://starbase.sysu.edu.cn/), miR-3681-5p shows a potential binding sites in HOXD-AS2 3’ UTR region. (B) The expression level of miR-3681-5p in GBM cells lines of U87 and LN229, as well as NHAs were detected by qRT-PCR. (C) The results of dual luciferase reporter assay in U87 cells showed that miR-3681-5p mimic inhibited luciferase activity in the wild type (WT) reporter containing miR-3681-5p potential binding sites in HOXD-AS2 3’ UTR region, but not in the reporter with mutated sequence (MUT). (D) The expression level of HOXD-AS2 in U87 and LN229 cells in the presence of miR-3681-5p mimic or inhibitor was detected by qRT-PCR. (E) The expression of miR-3681-5p in 92 GBM cancer tissues and adjacent normal tissues was detected by qRT-PCR. (F) Spearman correlation showed that there was a significant negative correlation between the expression of HOXD-AS2 and miR-3681-5p in GBM tumor tissues. The above data are the summary of the measurements of 3 independent experiments (mean ± standard deviation). **, P < 0.01, and ***, P < 0.001.

### 3.4. HOXD-AS2 maintains MALT1 expression via sponging miR-3681-5p

**Figure 4.**
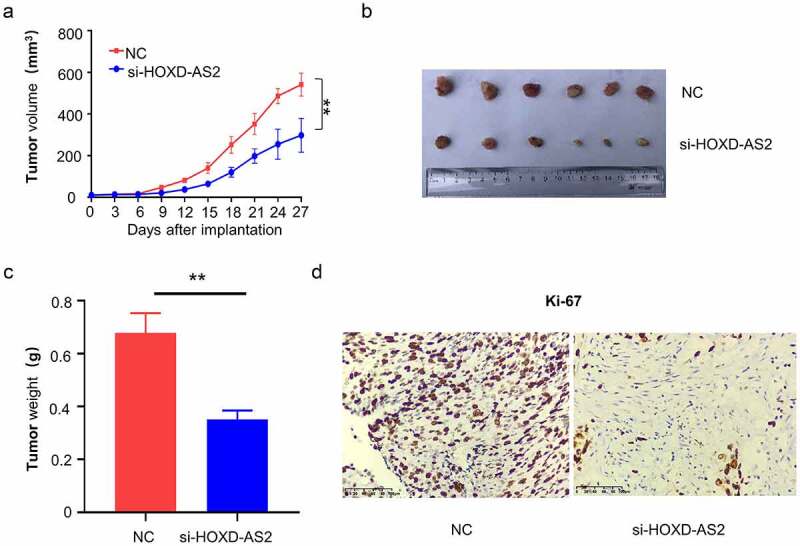
HOXD-AS2 maintains MALT1 expression via sponging miR-3681-5p

To identify the downstream target of miR-3681-5p, we searched miRTarBase database (http://mirtarbase.mbc.nctu.edu.tw/php/index.php) and found that, there was a potential binding site of miR-3681-5p in 3ʹ UTR region of MALT1 (Figure 4a). To validate the functional interaction between miR-3681-5p and MALT1 mRNA, we performed dual luciferase reporter assay using reporters containing miR-3681-5p binding site of MALT1 3ʹ UTR in U87 cells. The application of miR-3681-5p mimic inhibited the luciferase activity while the presence of miR-3681-5p inhibitor enhanced luciferase activity (Figure 4b), suggesting that miR-3681-5p negatively regulates MALT1 mRNA. qRT-PCR analysis showed that the expression of MALT1 in GBM cells (U87 and LN229 cells) was significantly higher than NHA cells (Figure 4c). Additionally, miR-3681-5p inhibitor elevated the expression of MALT1 while miR-3681-5p mimic suppressed MALT1 expression (Figure 4d). The effect of miR-3681-5p mimic and inhibitor on MALT1 expression was confirmed at protein level by western blot (Figure 4e). Importantly, we further analyzed the correlation of expressions between HOXD-AS2 and MALT1, as well as miR-3681-5p and MALT1, in 92 GBM tumor samples. Spearman correlation analysis revealed a significant negative correlation between miR-3681-5p and MALT1, and a positive correlation between MALT1 and the expression of HOXD-AS2 (figure 4f). Together, these results suggest that HOXD-AS2 maintains MALT1 expression by negatively targeting miR-3681-5p in GBM cells.

(A) Through miRTarBase (http://mirtarbase.mbc.nctu.edu.tw/php/index.php), there was a binding site of miR-3681-5p in noncoding region of MALT1 3’ UTR. (B) Luciferase reporter assay in U87 cells showed that miR-3681-5p mimic inhibited while miR-3681-5p inhibitor promoted luciferase activity in wild type reporter containing MALT1 3’ UTR. (C) The expression of MALT1 in GBM cell lines of U87 and LN229, as well as NHAs were detected by qRT-PCR. (D) Compared with NC, miR-3681-5p mimic down-regulated the expression of MALT1, whereas miR-3681-5p inhibitor up-regulated the expression of MALT1. (E) Western blotting showed that miR-3681-5p mimic down-regulated the protein level of MALT1, whereas miR-3681-5p inhibitor up-regulated MALT1. (F) There was a significant positive correlation between MALT1 and the expression of lncRNA HOXD-AS2, and a significant negative correlation between MALT1 and the expression of miR-3681-5p in tumor tissues. The above data are the summary of the measurements of 3 independent experiments (mean ± standard deviation). **, P < 0.01, and ***, P < 0.001.

### 3.5. miR-3681-5p mimic and MALT1 knockdown attenuate aggressiveness in GBM cells

**Figure 5.**
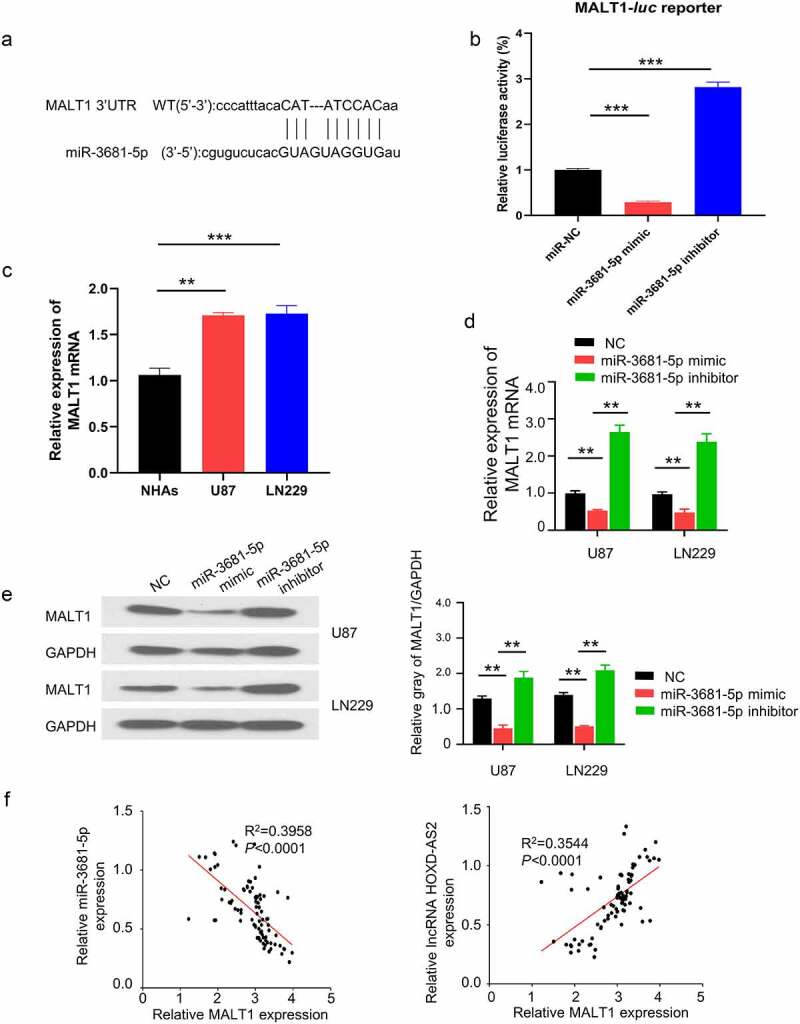
miR-3681-5p mimic or MALT1 knockdown attenuates aggressiveness in GBM cells

We next sought to validate the functional role of miR-3681-5p and MALT1 in GBM cells. Cells were transfected with HOXD-AS2 expression vector, HOXD-AS2 vector +miR-3681-5p mimic or HOXD-AS2 vector +MALT1 siRNA. As expected, HOXD-AS2 overexpression promoted cell proliferation. The cotransfection of miR-3681-5p mimic or MALT1 siRNA attenuated the effect of HOXD-AS2 overexpression (Figure 5a). Similarly, HOXD-AS2 overexpression promoted cell migration and invasion ability in both U87 and LN299 cells, which was attenuated by miR-3681-5p mimic and MALT1 siRNA (Figure 5b and 5c). These data indicate miR-3681-5p and MALT1 plays a functional role downstream of HOXD-AS2.

Cells were transfected with HOXD-AS2 expression vector, HOXD-AS2 vector +miR-3681-5p mimic or HOXD-AS2 vector +MALT1 siRNA. (A) CCK-8 assay indicated that the overexpression of HOXD-AS2 promoted the cell proliferation in U87 and LN229 cells, which was attenuated by miR-3681-5p mimic or MALT1 knockdown. (B) Wound-healing assay showed that that the overexpression of HOXD-AS2 significantly promoted the migration ability of cells, which was attenuated by miR-3681-5p mimic or MALT1 knockdown. (C) Transwell invasion assay showed that that the overexpression of HOXD-AS2 significantly promoted the invasion ability, which was attenuated by miR-3681-5p mimic or MALT1 knockdown. The above data are the summary of the measurements of 3 independent experiments (mean ± standard deviation). *, P < 0.05, and **, P < 0.01.

### 3.6. Silencing HOXD-AS2 inhibited GBM tumorigenesis in Xenograft model

**Figure 6.**
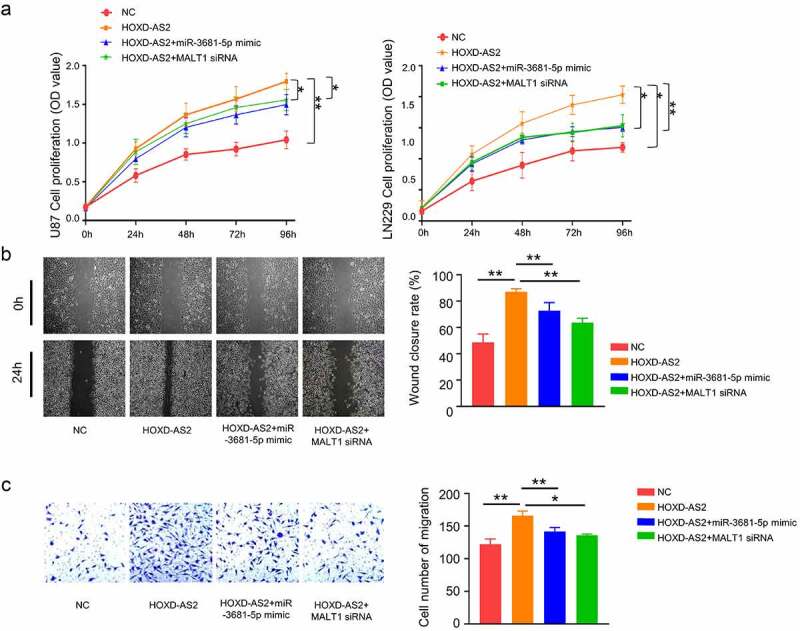
Downregulation of lncRNA HOXD-AS2 inhibits GBM tumor growth

Lastly, we aimed to validate the functional role in tumorigenesis of GBM. A total number of 12 nude mice were randomly assigned into two groups: NC group (n = 6, injected with U87 cells infected with siRNA control NC) and si-HOXD-AS2 group (n = 6, injected with U87 cells infected with siRNA for HOXD-AS2). The knockdown of HOXD-AS2 significantly suppressed the tumor growth, as revealed by the impaired increase of tumor volume and reduced tumor weight in si-HOXD-AS2 group (Figure 6a-c). We also performed immunohistochemical staining of Ki-67, a marker for cell proliferation. We found that the number of Ki-67 positive cells were decreased after the silencing of HOXD-AS2. Together, our results revealed that HOXD-AS2 is indispensable for cell proliferation and tumorigenesis of GBM cells.

(A) A total number of 12 nude mice were randomly assigned into two groups: NC group (n = 6, injected with U87 cells infected with siRNA control NC) and si-HOXD-AS2 group (n = 6, injected with U87 cells infected with siRNA for HOXD-AS2). Tumor volume was monitored every three days for 4 weeks. On day 28, mice were sacrifice and tumor in each mouse was dissected (B), and the tumor weight was recorded in each group (C). (D) Immunohistochemical staining of Ki-67 showed decreased number of Ki-67 cells in tumors from si-HOXD-AS2 group. Bar represents 100 μm. **, P < 0.01.

## 4. Discussion

Our study explored the roles of lncRNA HOXD-AS2 in glioblastoma cell proliferation, migration and invasion by regulating the miR-3681-5p/MALT1 signaling pathway. Our experiments demonstrated that HOXD-AS2 expression was significantly upregulated in GBM tumor tissues and cell lines. Its knockdown inhibited GBM cell proliferation, migration and invasion, and promoted cell apoptosis. Our results suggest that HOXD-AS2 could act as a sponge for miR-3681-5p to maintain MALT1 expression and support the aggressive phenotype of GBM cells, since miR-3681-5p overexpression or MALT1 knockdown attenuated the effect of HOXD-AS2 overexpression in GBM cells.

An increasing number of studies have revealed the implications of lncRNAs in the progression of GBM, which may serve as biomarker for diagnosis and prognosis. However, the functional roles of most lncRNAs in GBM are unclear. Consistent with a previous study [9], we showed that the expression level of HOXD‐AS2 in GBM tissues was higher than normal tissues. We further demonstrated that patients with high HOXD-AS2 expression are associated with a lower overall survival rate. These data suggest that HOXD-AS2 may be involved in the progression of GBM. Consistently, down-regulating the expression of HOXD-AS2 in GBM cells not only impairs cell proliferation, migration and invasion, but also attenuates tumorigenesis *in vivo*.

It has been demonstrated that lncRNAs can act as miRNA sponges to prevent the binding of miRNAs with their target mRNAs, releasing mRNAs for translation [21,22]. This function adds another layer of complexity the miRNA-target interaction network [23]. According to a previous study [24], lncRNA cancer susceptibility candidate 2 (CASC2) could modulate glioma growth and resistance to Temozolomide through phosphatase and tensin homolog (PTEN) pathway by targeting miR-181a. It has also been reported that lncRNA H19 was up-regulated in glioma tissues [25], which interacts with miR-140 to regulate glioma growth by targeting Inhibitor of Apoptosis-Stimulating Protein of p53 (iASPP) [26]. These studies encouraged us to explore if lncRNA HOXD-AS2 could connect with miRNA(s) in glioma to regulate the aggressive phenotype. Through the search of miRNA database, we discovered that miR-3681-5p had a potential binding site in HOXD-AS2 3ʹ UTR region and there was also a binding site between miR-3681-5p and MALT1 3ʹ UTR. We further confirmed that miR-3681-5p expression level in GBM cell lines of U87 and LN229 was significantly lower than that in normal cells, while the expression of MALT1 in GBM cell was significantly upregulated. The results of luciferase reporter assay in U87 cells showed that compared with miR-NC, miR-3681-5p mimic inhibited luciferase activity of reporter containing MALT1 3ʹ-UTR binding site. Besides, the overexpression of miR-3681-5p down-regulated the expression of MALT1, whereas miR-3681-5p inhibitor up-regulated MALT1 expression. Spearman correlation further showed that there was a significant positive correlation between MALT1 and the expression of HOXD-AS2 in GBM tumor samples, and the expression of miR-3681-5p in tumor tissues showed a negative correlation with MALT1 and HOXD-AS2. Taken together, our data strongly suggest that lncRNA HOXD-AS2 maintains MALT1 expression via sponging miR-3681-5p.

Based on our results, the overexpression of miR-3681-5p and the knockdown of MALT1 showed similar effect on GBM cells, which both attenuated the aggressiveness of GBM cells. A previous study showed that MALT1 acts as a scaffold protein that recruits TRAF6 to the IKK complex to activate NF-κB after EGF stimulation [27]. MALT1 plays an important role in activating NF-κB signaling pathway, and the activation of NF-κB is very common in GBM. Therefore, MALT1 has been recognized as an oncogene in cancer progression [28,29]. The potential regulatory mechanism has been explained [30]. Consistent with this notion, our results not only showed the upregulation of MALT1 in GBM tumors, but also implicated MALT1 as a downstream factor mediating the effect of HOXD-AS2. Importantly, *in vivo* experiments further confirmed that the knockdown of HOXD-AS2 impaired the tumor growth. Therefore, our study provides novel evidence that lncRNA HOXD-AS2 is indispensable for GBM tumor growth by regulating miR-3681-5p/MALT1 axis.

## 5. Conclusion

Our study showed that silencing lncRNA HOXD-AS2 significantly inhibits the cell proliferation and tumorigenesis of glioma cells, and HOXD-AS2 can regulate the expression of MALT1 by serving as a molecular sponge of miR-3681-5p. Future work is required to study the mechanism by which HOXD-AS2 is upregulated in GBM cells, which can provide novel insights into therapeutic intervention for GBM treatment.

## Highlights

- LncRNA HOXD‐AS2 is upregulated in Glioblastoma tumor and cell lines.
- Silencing HOXD‐AS2 attenuates the aggressiveness of GBM cells *in vitro* and *in vivo*
- HOXD‐AS2 targets miR-3681-5p to negatively regulate its expression.
- miR-3681-5p/MALT1 axis contributes to the oncogenic function of HOXD‐AS2
